# The effect of Omega-3 polyunsaturated fatty acid supplementation on exercise-induced muscle damage

**DOI:** 10.1186/s12970-020-00405-1

**Published:** 2021-01-13

**Authors:** Yvoni Kyriakidou, Carly Wood, Chrystalla Ferrier, Alberto Dolci, Bradley Elliott

**Affiliations:** 1 Translational Physiology Research Group, School of Life Sciences, College of Liberal Arts and Sciences, University of Westminster, London, W1W 6UW UK; 2 School of Sport, Rehabilitation and Exercise Sciences, University of Essex, Essex, UK

**Keywords:** Omega-3, Supplementation, Ergogenic aid, Eccentric exercise, Muscle damage, EIMD, DOMS, Recovery, Inflammation, Performance

## Abstract

**Background:**

Exercise-induced muscle damage (EIMD) results in transient muscle inflammation, strength loss, muscle soreness and may cause subsequent exercise avoidance. Omega-3 (n-3) supplementation may minimise EIMD via its anti-inflammatory properties, however, its efficacy remains unclear.

**Methods:**

Healthy males (*n* = 14, 25.07 ± 4.05 years) were randomised to 3 g/day n-3 supplementation (N-3, *n* = 7) or placebo (PLA, *n* = 7). Following 4 weeks supplementation, a downhill running protocol (60 min, 65% V̇O_2_max, − 10% gradient) was performed. Creatine kinase (CK), interleukin (IL)-6 and tumour necrosis factor (TNF)-α, perceived muscle soreness, maximal voluntary isometric contraction (MVIC) and peak power were quantified pre, post, and 24, 48 and 72 h post-EIMD.

**Results:**

Muscle soreness was significantly lower in N-3 vs PLA group at 24 h post-EIMD (*p* = 0.034). IL-6 was increased in PLA (*p* = 0.009) but not in N-3 (*p* = 0.434) following EIMD, however, no significant differences were noted between groups. Peak power was significantly suppressed in PLA relative to pre-EIMD but not in N-3 group at 24 h post-EIMD. However, no significant difference in peak power output was observed between groups. MVIC, CK and TNF-α were altered by EIMD but did not differ between groups.

**Conclusion:**

N-3 supplementation for 4 weeks may successfully attenuate minor aspects of EIMD. Whilst not improving performance, these findings may have relevance to soreness-associated exercise avoidance.

**Supplementary Information:**

The online version contains supplementary material available at 10.1186/s12970-020-00405-1.

## Background

The recovery from vigorous athletic performance concerns many groups of people, from high performance athletes to recreationally active individuals. Eccentric exercise, especially novel or high-force eccentric protocols, can produce substantial muscle fibre damage [1, 2]. Such vigorous-intensity exercise may lead to exercise-induced muscle damage (EIMD) [3]. Symptoms of EIMD include pain, swelling, muscle strength and power loss, reduced range of motion (ROM), delayed onset muscle soreness (DOMS) and impaired recovery [4, 5]; resulting in impairment of exercise performance [6]. Functionally, muscle strength is reduced by ~ 20 to 50% immediately and post-exercise can take between 2 to 7 days to fully recover [7]. Systemically, EIMD is paralleled by an inflammatory response involving many mediators, such as interleukin − 1 receptor antagonist (IL-1ra), interleukin (IL)-6, IL-10 and acute phase proteins [8], and a release of muscle specific creatine kinase [1, 9]. Strategies to reduce muscle damage and inflammation following EIMD can therefore be of use to individuals interested in increasing their rate of recovery and maintaining performance.

Omega-3 polyunsaturated fatty acids (n-3 PUFA) have a double bond that is closest to the methyl terminus (−CH_3_) of the acyl chain [10]. N-3 PUFA are incorporated into phospholipids, altering cell membranes, which typically contain a high proportion of arachidonic acid (AA). This results in increased accumulation of eicosapentaenoic acid (EPA) and docosahexaenoic acid (DHA) and at the parallel decline of AA; and potentially blunting reactive oxygen species (ROS) and inflammatory cytokine production [10]. Anti-inflammatory mediators derived from n-3 PUFA and its main bioactive fatty acids, such as EPA and DHA, have been recognised along with their mechanism of their action [10]. It has been suggested that n-3 PUFA may prove a viable strategy to attenuate muscle inflammation and improve functional recovery following high-intensity exercise [11]. One of the connections between n-3 PUFA and muscle inflammation is via down-regulation of pro-inflammatory cytokines, such as TNF-α and IL-6, reduced production of AA and ROS, consequently, resulting in a decrease in the inflammatory response [12].

Increasing evidence suggests that n-3 supplementation impairs pro-inflammatory cytokines and ROS production, and hence may show a direct relationship between intense exercise recovery and markers of inflammation [12, 13]. Although animal studies have shown mixed results when evaluating the efficacy of n-3 supplementation on muscle damage, exercise metabolism and exercise performance; human studies have demonstrated that physiological parameters that are linked to improved physical performance and oxygen utilisation, such as blood flow during exercise, can be augmented by dietary n-3 PUFA [11, 14]. Tarbinian et al. [15], Jouris et al. [16] and Jakeman et al. [17] have shown a pain reduction following EIMD with n-3 supplementation. A recent meta-analysis [18] also concluded that n-3 supplementation could alleviate DOMS after eccentric exercise. Additionally, Atashak et al. [19] reported substantial reduction in CK and in C-reactive protein after lower body resistance exercise following 1 week of 540 mg EPA and 360 mg DHA. Further, other studies [20, 21] have demonstrated that n-3 supplementation has a positive effect on eccentric exercise protocols by reducing the concentrations of IL-6 and TNF-α. However, mixed results have been reported to date, with others [22, 23] observing no effect of n-3 supplementation on exercise-induced inflammatory and muscle damage markers, and functional markers, such as maximal voluntary contraction (MVC) and DOMS.

It remains unclear whether n-3 supplementation has any beneficial effect in blunting the effects of EIMD, either by increasing the rate of recovery of functional performance, by reducing circulating pro-inflammatory cytokines, or both. Due to this lack of clarity in the literature, the aim of the current study was to add evidence by assessing the effect of n-3 supplementation on EIMD following a downhill running bout. It was hypothesised that 3 g of n-3 supplementation for 4 weeks would attenuate muscle inflammation following EIMD which subsequently would decrease the recovery time, and thus improve exercise performance.

## Methods

### Participants

Ethical approval was obtained by the College of Liberal of Arts and Sciences Research Ethics Committee, University of Westminster (ETH1617–0182). All work herein conforms to the standards set by the Declaration of Helsinki of 1975. Written informed consent was obtained from all participants prior to their participation.

A total of 23 healthy, physically active males (self-reported: 4–5 times weekly structured exercise) aged 18–35 years of age were recruited to participate in this experimental study. Following withdrawals (*n* = 9; inability to attend all visits, injury or illness outside of trials or inability to complete downhill protocol), 14 participants (25.07 ± 4.05 years of age) completed the protocol and are included in the analysis below.

Participants were required to refrain from any structured exercise for 48 h, and from alcohol and caffeine 24 h prior to baseline visit and EIMD protocol. They were also asked to refrain from eccentric strenuous exercise during the 5 weeks of the study as well as in the following 72 h after the muscle-damaging exercise bout. Exclusion criteria included age (outside 18–35 age range), smoking, sex, taking any medication (e.g. non-steroidal anti-inflammatory drugs), and consuming fish oil supplements < 6 months prior to commencing the study and the presence of any known immune, cardiovascular or metabolic disease. To further confirm participants were free from upper respiratory tract infections, they completed an illness-specific questionnaire (WURSS-21) [24]. Additionally, participants were free from any pain or injury as determined by the Physical Activity Readiness Questionnaire (PAR-Q) pre-exercise participation screening. Participants were also excluded if they regularly undertook downhill running or eccentric exercise (e.g. resistance exercise, squats) as part of their normal training < 6 months prior to commencing the study.

### Experimental design

All participants were required to attend the human performance laboratory in the morning on 5 occasions. During visit 1, in an overnight fasted-state, participants performed baseline measurements which included anthropometric measurements, a urine sample and a venous blood sample. Perceived muscle soreness, maximal voluntary isometric contraction (MVIC) on the leg and anaerobic peak power via Wingate test were determined as indirect markers of muscle damage, described fully below.

Following baseline measurements, participants performed a treadmill V̇O_2_max test (HP Cosmos Mercury 4.0, Nussdorf-Traunstein, Germany) with expired gases analysed by an on-line breath-by breath system (Cortex Metalyser 3B, Biophysik, Leipzig, Germany). The Metalyser was calibrated according to manufacturer’s guidelines prior to each test. Following completion of the V̇O_2_max test, a 6-min running speed (V_test_) at 65% V̇O_2_max verification was performed on a downhill run at − 10% gradient, as we have previously reported [25]. After baseline testing, participants were single-blind randomised to either N-3 (3 g/day of n-3 PUFA) or PLA (placebo) group by a computer-generated block randomization in advance (http://www.randomization.com).

**Fig. 1:**
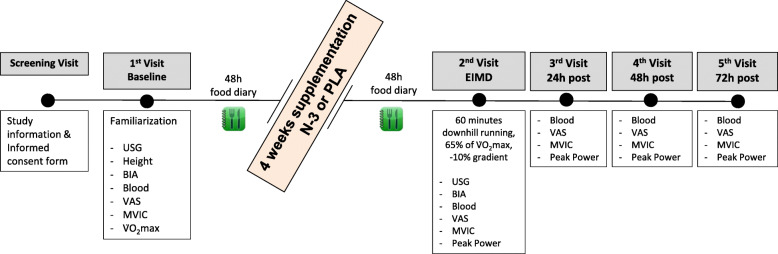
Schematic of experimental procedures: USG, urine specific gravity; BIA, bioelectrical impedance analysis; VAS, visual analogue scale for delayed-onset muscle soreness; MVIC, maximal voluntary isometric contraction; V̇O_2_max, maximal oxygen consumption; N-3, omega-3 supplementation group; PLA, placebo group; EIMD, exercise-induced muscle damage. 2nd visit combines both pre and post measurements, immediately prior and following EIMD stimulus, respectively. A further Wingate test was added on 2nd visit and all follow up visits

Two weeks before beginning testing, participants filled a health questionnaire (WURSS-21) on each of the 14 days preceding trial to ensure that they were free from common cold symptoms before testing. In visit 2, participants reported to the laboratory at 07:00 am having fasted overnight and performed the EIMD protocol (downhill running; 60 min at 65% V̇O_2_max with a − 10% gradient). All above measurements were repeated prior to- and immediately-post the EIMD trial. One day before the visit participants were asked to consume water based on their body mass (5 mL/kg) [25] before they reported to the laboratory to ensure adequate hydration before exercise. Identical follow up assessments, except urine sample, were repeated at visits 3-to-5 (24, 48 and 72 h post-EIMD), during which participants were in a non-fasted state. An overview of the study design is presented in Fig. 1.

### Hydration status

Urine sample was collected at baseline and pre-EIMD to assess participants’ hydration status. Hydration was verified by checking that urine specific gravity (USG) (Atago MASTER-SUR/Na refractometer, Atago Co., Ltd. Tokyo, Japan) upon arrival was between 1.001 and 1.029 [26]. Urine colour was also checked by using the validated urine colour chart (1–8 scale) [26].

### Anthropometric measurements

Height (to nearest 0.1 cm) was measured using a wall-mounted “Harpenden” stadiometer (Holtain Ltd., Crymych, Wales, UK) fitted with a high speed Veeder-Root counter (Veeder-Root, Elizabethtown, NC, USA) with participants stood in bare feet, heels together with their shoulders and buttocks in contact with the stadiometer. Body weight (to nearest 0.1 kg), BMI and body fat % (to nearest 0.1%) were measured using BIA (Tanita SC-330ST, Tokyo, Japan) with participant being fasted and with an empty bladder.

### Supplementation

Omega-3 supplementation consisted of 3 gelatine-coated capsules per day (1 consumed in the morning, 1 at lunch and 1 in the evening), each containing 1040 mg of n-3 PUFA (715 mg of EPA and 286 mg DHA) per capsule (Maximum Strength Pure Fish Oil, Nature’s Best, UK) for a total of 3900 mg of fish oil daily, containing 3 g of n-3 PUFA (2145 mg of EPA and 858 mg DHA) per day for a period of 4 weeks. Whilst commonly reported side effects of n-3 supplementation, such as unpleasant taste, heartburn, gastrointestinal discomfort and headache are usually mild [27], the amount of n-3 provided is in line with the nutritional recommendations as part of a normal diet and does not cause any harm or side effects. Daily supplementation of up to about 5 g/day of n-3 PUFA in a long-term consumption is considered safe by the European Food Safety Authority (EFSA) [28]. Longer duration or high doses may affect immune function due to suppression of inflammatory response [29]. High doses also might increase bleeding time by reducing platelet aggregation [29]. The placebo group received 3 × 600 mg capsules per day of collagen (Troo Healthcare, Colchester, UK), consumed in a matching pattern. Participants were only given 1 week of capsules at a time. Initially written reminders were sent on a daily basis to ensure supplementation practices were maintained consistent throughout the day. Participants’ compliance also verified by weekly written and oral reminders, counting remaining capsules at the end of each week and issuing of future weeks capsules took place. Further, participants were asked to guess what group they were at the conclusion of testing with 2 of 7 in placebo and 5 of 7 in N-3 group correctly guessing the supplementation group.

### Diet and activity control

Participants were requested to maintain their usual diet and physical activity throughout the study. A 48-h food diary (including 1 day of the week and one weekend day) was provided to record all foods and drinks consumed prior to the supplementation period starting. Following 4 weeks of supplementation, participants completed a second 48-h food diary in the 2 days prior to the EIMD trial. Written and oral reminders were also provided on a regular basis to ensure diet and exercise practices were maintained consistent throughout the study.

Food diaries were analysed using Nutritics® to quantify total energy intake, macronutrients (carbohydrates, protein, fatty acids), n-3 and n-6 PUFA before and after the supplementation period. In addition, at the start of the supplementation period all participants were provided with a food list with foods low (< 250 mg per serving), moderate (~ 250 mg per serving) and high (> 500 mg per serving) in omega-3 fatty acids to prevent increasing their omega-3 intake through diet. Cut-off points were used by USDA SR-21 (2008) [30].

### EIMD protocol

Following a 3-min warm up, participants ran for 60 min at the individualized predetermined V_test_ at − 10% gradient. Heart rate (HR) and rating of perceived exertion (RPE), Borg 6–20 scale [31] were recorded throughout the trial every 10 min. A 60-s sample expired of gases were collected at 20 and 40 min of trial and analysed by an on-line breath-by breath system for V̇O_2_ to ensure participants were running at 65% V̇O_2_max. Water was provided ad libitum every 15 min whilst running. Immediately after the muscle-damaging bout participants sat and a blood sample was collected (post-EIMD). Participants then rated their perceived muscle soreness, and MVIC and Wingate test were performed to assess participants’ post-EIMD strength and power output, respectively.

### Venous plasma

A total of 12 mL of venous blood was collected at each time point in two 6 mL vacutainer tubes (K_2_ EDTA and lithium-heparin; BD, Oxford, UK). Haematocrit with capillary method using a micro-hematocrit reader (Hawksley & Sons Ltd., Lancing, UK) and haemoglobin concentration using a photometer (Haemocue, Sheffield, UK) were both analysed immediately on heparinized whole blood in triplicate. Subsequently, concentration of plasma markers was adjusted for plasma volume changes with the method of Dill and Costill [32]. The remaining whole blood was spun (Hettich Universal 320 R, Germany) at 5000 rpm for 10 min at 4 °C, with plasma aliquoted and frozen at − 80 °C.

Circulating CK activity was measured using a clinical chemistry analyser (Werfen ILab Aries, Italy). CK activity was determined using kinetic spectrophotometry at 340 nm with a minimum detection limit of 3 U/L, an undiluted linearity up to 900 U/L. Coefficient of variation (CV) was within run < 1.2%, total < 2.5%. All samples and standards were analysed in duplicate.

Aliquots of plasma were analysed for plasma IL-6 and TNF-α concentration by enzyme-linked immunosorbent assay (ELISA) DuoSet kits and ancillary reagent packs (IL-6 DY206, TNF-α, DY210, consumables DY008, R*&*D Systems, USA) in duplicate, according to manufacturer’s instruction. Plates were read at 450 nm and blanked to 590 nm.

### Perceived muscle soreness

Muscle soreness was self-rated by participants on a 10-point-validated visual analogue scale (VAS) indicating on a line from 0 (no pain) to 10 (extreme pain) [33], during a wall squat with thighs parallel to the floor at 90^0^ degrees.

### Maximal voluntary isometric contraction

MVIC was assessed on a dynamometer (Globus Kineo 7000, Italy). The chair was adjusted so that the leg pad was placed on the lower part of the tibialis anterior and the pivot was located on the lateral epicondyle of the right leg. Maximal force was measured at an angle of 60^o^ leg extension. Peak force was determined by the average of four maximal isometric contractions lasting 3–5 s. The contraction time was recorded by an experimenter.

### Peak power

Participants performed a 10 s Wingate test on a cycle ergometer (Monark Ergomedic 894E, Sweden), fixed with an optical sensor (OptoSensor 2000™, Sport Medicine Industries, USA) with the data obtained by the Monark Anaerobic Test Software. Participants cycled seated during the sprint protocol, with a resistance equal to 7.5% of their body weight. Participants were verbally encouraged throughout the test.

### Statistical analysis

Normal distribution of all data was performed by the Shapiro-Wilk Test. Following Levene’s test of equality of variance, baseline characteristics, dietary and hydration data were compared between groups using a two-tailed independent samples t-test. The examination of the effect of the n-3 supplementation on plasma CK activity, IL-6, TNF-α and DOMS was performed by non-parametric tests, as these variables did not follow normal distribution. Mann-Whitney U test was performed to examine differences between N-3 and PLA group at each time point. A Freidman test was used to determine the main effect of time within-group and post hoc with Wilcoxon Signed Rank tests (using a Bonferroni adjusted alpha value) was run where a significant time was identified. MVIC and peak power data met all assumptions required for normality and were analysed using a two-way mixed between-within participant repeated measures analysis of variance (ANOVA). Bonferroni-adjust pairwise comparisons post hoc analysis was used where needed to examine within subject differences. Values were expressed as mean ± SD for data from parametric tests and as median and interquartile range for data from non-parametric tests. Statistical significance was accepted as *p* < 0.05. Effect size was calculated using methods proposed by Cohen [34], with effect sizes considered small (0.2), medium (0.5) or large (0.8). Statistical analyses were performed using SPSS 25 software (IBM SPSS, NY, USA). All figures were generated in GraphPad Prism (Version 8, GraphPad).

#### Power calculation

The sample size was estimated from a sample calculation (G*Power 3.1) with an alpha level of 0.05, a power (1-β) of 0.80 and a medium effect size of 0.5 and suggests *n* = 12 in total would be sufficient.

## Results

### Descriptive characteristics

The physical characteristics of participants completed EIMD are presented in Table 1. There was homogeneity in all characteristics of participants between groups.

**Table 1:** Physical characteristics of participants completed EIMD, independent sample t-test comparison between N-3 and Placebo group

	Total (***n*** = 14)	N-3 (***n*** = 7)	PLA (***n*** = 7)	***P***-value
**Age (years)**	25.07 (± 4.05)	25.57 (± 4.18)	24.57 (± 4.24)	0.66
**Weight (kg)**	73.04 (± 9.82)	69.34 (± 10.93)	76.74 (± 7.59)	0.17
**Height (cm)**	179.59 (± 10.24)	174.60 (± 11.64)	184.59 (± 5.77)	0.06
**BMI (kg/m**^**2**^**)**	22.60 (± 1.80)	22.63 (± 1.41)	22.59 (± 2.24)	0.97
**Body fat (%)**	10.75 (± 4.07)	10.40 (± 4.29)	11.10 (± 4.15)	0.76
**V̇O**_**2**_**max (ml/kg/min)**	62.43 (± 11.77)	65.11 (± 11.08)	59.74 (± 12.66)	0.41

Values are expressed as mean ± SD. N-3, omega-3 group; *PLA* placebo group, *BMI* body mass index, *V̇O*_*2*_*max* maximal oxygen consumption

Descriptive characteristics in dietary data at baseline (before supplementation) of participants from both groups are presented in Table 2. An independent-samples t-test was conducted to compare energy, macronutrients and n-6/n-3 ratio between groups, and Mann-Whitney U test was performed to compare n-3 intake [N-3, Md = 7.57 (53.00); PLA, Md = 7.43 (52.00), U = 24.00, Z = − 0.064] and n-6 intake [N-3, Md = 8.21 (57.50); PLA, Md = 6.79 (47.50), U = 19.50, Z = − 0.640] between groups. There was no significant difference in food intake (*p* > 0.05) at baseline between N-3 and PLA group (Table 2).

**Table 2:** Dietary data at baseline between N-3 and PLA groups

	Total (***n*** = 14)	N-3 (***n*** = 7)	PLA (***n*** = 7)	***P***-value
**Energy (Kcals)**	2580.64 (± 789.39)	2664.71 (± 414.35)	2496.57 (± 1077.93)	0.707
**CHO (g)**	296.64 (± 83.62)	319.71 (± 59.15)	273.57 (± 102.02)	0.321
**Protein (g)**	109.11 (± 30.21)	108.64 (± 23.20)	109.57 (± 37.94)	0.957
**Fat (g)**	94.99 (± 46.12)	89.48 (± 28.06)	100.49 (± 61.23)	0.673
**n-3 (g)**^**a**^	1.56 (± 1.43)	1.41 (± 1.20)	1.70 (± 1.72)	0.949
**n-6 (g)**^**a**^	9.47 (± 7.42)	9.85 (± 6.59)	9.09 (± 8.69)	0.522
**n-6/n-3 ratio**	8.20 (± 4.91)	9.18 (± 4.81)	7.21 (± 5.18)	0.476

Values are expressed as mean ± SD. *N-3* omega-3 group, *PLA* placebo, *CHO* carbohydrates, *n-3* omega-3 fatty acids, *n-6* omega-6 fatty acids. ^a^indicate Mann-Whitney U test

Descriptive characteristics in dietary data after the supplementation period of participants from both groups are presented in Table 3. An independent-samples t-test was conducted to compare energy, macronutrients, n-3 and n-6 intake between groups. There was a significant difference in n-3 intake with N-3 group showing a higher n-3 intake post supplementation. However, there was no significant difference in any other food intake (*p* > 0.05) between groups (Table 3).

**Table 3:** Dietary data post supplementation (+ 3 g of n-3 supplementation) of participants completed EIMD, independent sample t-test comparison between N-3 and Placebo group

	Total (***n*** = 14)	N-3 (***n*** = 7)	PLA (***n*** = 7)	***P***-value
**Energy (Kcals)**	2574.57 (± 928.64)	2653.43 (± 794.99)	2495.71 (± 1105.43)	0.765
**CHO (g)**	305.64 (± 134.93)	293.50 (± 68.18)	317.78 (± 185.62)	0.751
**Protein (g)**	109.79 (± 32.36)	104.06 (± 26.08)	115.52 (± 38.89)	0.529
**Fat (g)**	92.84 (± 37.37)	104.33 (± 38.87)	81.35 (± 34.74)	0.266
**n-3 (g)**	2.82 (± 1.92)	3.87 (± 1.90)	1.78 (± 1.34)	0.036*
**n-6 (g)**	8.47 (± 5.41)	8.65 (± 5.83)	8.29 (± 5.42)	0.906
**n-6/n-3 ratio**	4.65 (± 3.80)	3.19 (± 3.18)	6.12 (± 4.03)	0.157

^*^Significant level, *p* < .05; Values are expressed as mean ± SD. *N-3* omega-3 group, *PLA* placebo, *CHO* carbohydrates, *n-3* omega-3 fatty acids, *n-6* omega-6 fatty acids

In addition, an independent-samples t-test was conducted to compare participants’ hydration status before EIMD between N-3 and PLA group. There was no significant difference in hydration status [N-3, *n* = 7, (M = 1.015, SD = 0.007) and PLA, *n* = 7, (M = 1.012, SD = 0.007); t (12) = 0.72, *p* > 0.05, two-tailed] between groups.

### Blood markers

**Fig. 2:**
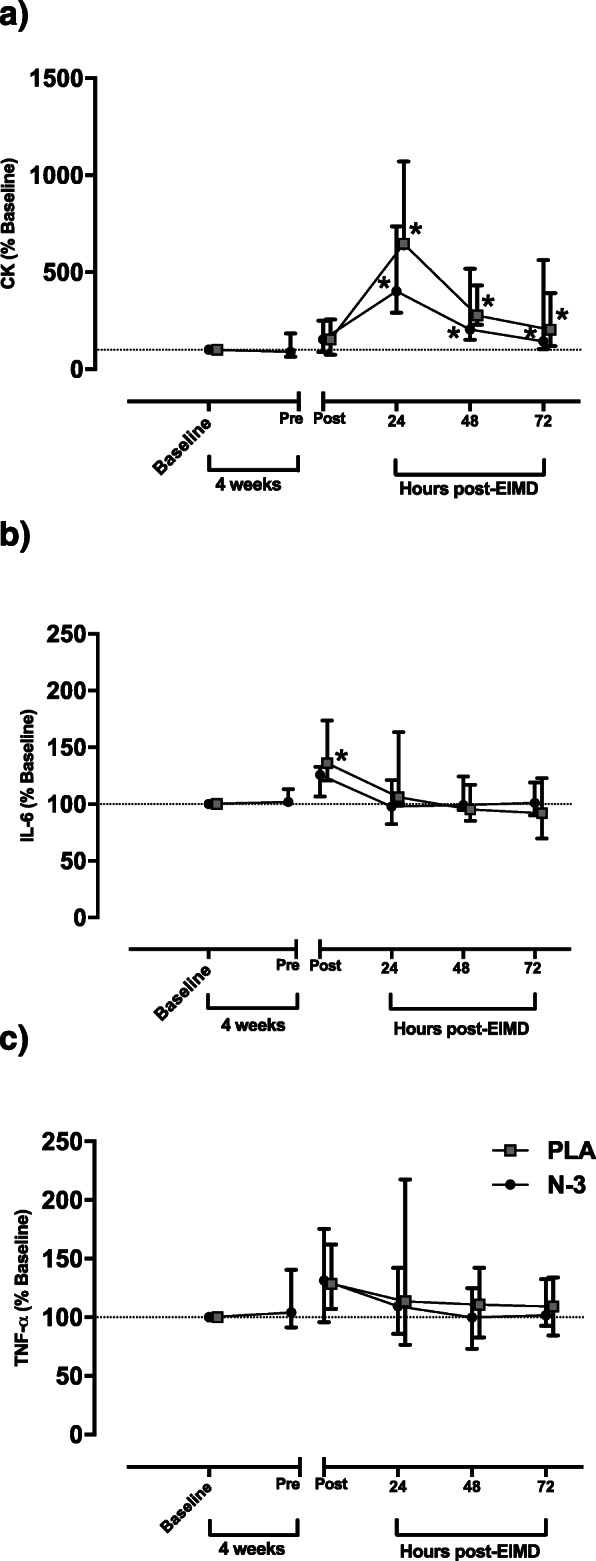
Plasma markers of muscle damage and inflammation as a function of time. **a** CK (% change from baseline), **b** IL-6 (% change from baseline), and **c** TNF-α (% change from baseline). Data shown as medians, error bars indicate interquartile range. Dashed horizontal line indicates 100% (baseline). * indicates significant difference from baseline. Time matched data points offset horizontally to enhance clarity

Friedman’s ANOVA suggested an effect of time on CK activity for both PLA and N-3 groups (both *p* < 0.001), with post hoc testing suggesting both groups were increased at 24, 48 and 72 h relative to baseline (*p* < 0.05, *r* = 0.63 indicating a medium effect size for all three time points; Fig. 2.

A). However, there was no significant difference between PLA and N-3 at any timepoint.

Mann-Whitney U test was used to compare plasma IL-6 concentration between N-3 and PLA group. There was no significant difference between groups at any time point. Nevertheless, Friedman’s ANOVA revealed plasma IL-6 did not change over time in N-3 group (*p* = 0.434) but did change in PLA group (*p* = 0.009). Post hoc testing suggested IL-6 was elevated in the PLA group at immediately post-EIMD relative to baseline (*p* < 0.05, *r* = 0.61 indicating a medium effect size) but no other time points (Fig. 2)

B). Mann-Whitney U test was performed to compare plasma TNF-α concentration between groups. Plasma TNF-α did not differ with time in either PLA (*p* = 0.274) or N-3 group (*p* = 0.345; Fig. 2c).

### Functional measurements

**Fig. 3:**
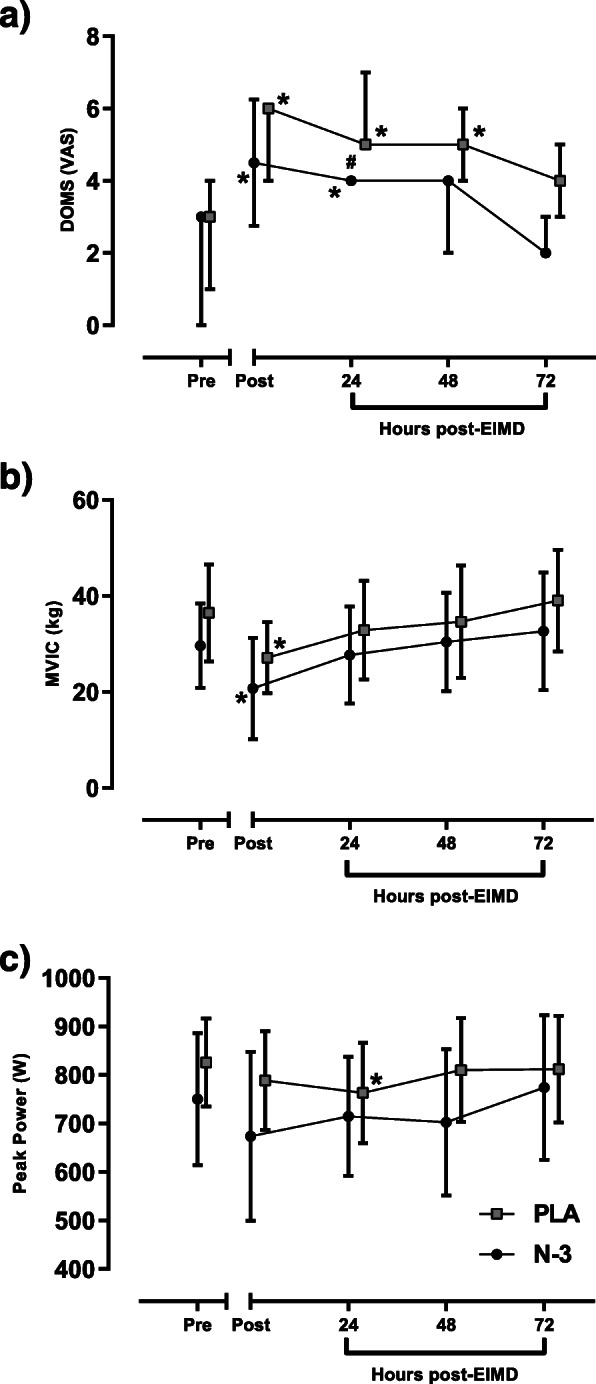
Muscle functional measures prior (pre) and following (post – 72 h) EIMD. **a** DOMS, data indicates median, error bars interquartile range. Both **b** MVIC (Kg), and **c** peak power (W) data indicate means, error bars standard deviation. * indicates significant difference from Pre timepoint, # indicates difference between groups at timepoint indicated. Time matched data points offset horizontally to enhance clarity

Mann-Whitney U test was run to compare perceived muscle soreness between N-3 and PLA group at each time point. There was a statistically significant difference in DOMS between groups at 24 h post-EIMD, with PLA showing a higher muscle soreness compared to N-3 group (*p* = 0.034) with a medium effect size (*r* = 0.56). Friedman’s test suggested DOMS significantly differed both within the N-3 and PLA group (*p* < 0.001). Pairwise comparisons suggested that N-3 group had elevated DOMS immediately post (*r* = 0.57) and at 24 h post-EIMD (*r* = 0.59) relative to pre (all *p* < 0.05), whilst the PLA group maintained DOMS for longer, being elevated immediately post (r = 0.64), and at both 24 and 48 h post-EIMD (*r* = 0.6) relative to pre (all *p* < 0.05; Fig. 3a).

No group x time interaction was noted for MVIC (repeated measures ANOVA, *p* = 0.813) or a main effect of group (*p* = 0.338). A significant main effect for time was observed for MVIC leg strength (*p* = 0.011, η_p_^2^ = 0.813). This result suggests a large effect size with MVIC suppressed relative to pre in both N-3 and PLA groups (N-3 = 29.6 (±8.8) kg vs 20.8 (±10.6) kg, PLA = 36.5 (±10.2) kg vs 27.1 (±7.4) kg; both *p* < 0.05) immediately post-EIMD, but no other time points (Fig. 3b).

No group x time interaction was noted for peak power (repeated measures ANOVA, *p* = 0.514) or a main effect of group (*p* = 0.310). A significant main effect for time was observed for peak power (*p* = 0.014, η_p_^2^ = 0.841). This result suggests a large effect size with post hoc testing suggesting no change in peak power following EIMD in N-3 group, but a suppression in peak power in PLA group at 24 h relative to pre [pre-EIMD = 825.6 (±90.7), 24 h = 763.0 (±103.1) W, *p* < 0.05] (Fig. 3c).

## Discussion

Results presented here suggest that 4 weeks supplementation with 3 g/day of n-3 PUFA offsets the EIMD induced pain response following a single bout of high intensity exercise. Whilst a decreased peak power output in PLA group at 24 h following EIMD and a blunted time response in pro-inflammatory marker IL-6 was witnessed in the N-3 group immediately post-EIMD, no between group differences were found. Although findings suggest decreased MVIC and increased plasma CK following EIMD, no difference was observed between groups, overall suggesting minimal positive gain in exercise performance with n-3 supplementation.

### Blood markers

Our data show a significant increase of CK activity following EIMD before returning to baseline in both groups, mirroring those previously reported [9, 35]. Conversely, Bloomer et al. [20] reported no significantly increased CK activity following eccentric exercise. This finding is in agreement with the findings by Atashak et al. [19], another study [36] with a similar exercise protocol, following a 40 min downhill running, and Tsuchiya et al. [23] after an eccentric protocol with a similar dose but longer supplementation period (8 weeks).

The data presented here showed a large degree of variability in circulating CK (from 65 to 4939 U/L) which is in accordance with previous research [37]. Hence, CK alone might not be an accurate reflection of the degree of muscle damage following eccentric exercise [38] due to large inter-individual variability in response with a range from 236 to 25,244 IU/L [1]. Therefore, the results of the efficacy of the n-3 supplementation on indirect muscle damage biomarkers, such as CK, following maximal exercise performance may be inconsistent due to variability alone, and such markers should not be considered in isolation.

Plasma IL-6 concentration peaked immediately post-EIMD for the PLA group. This peak of plasma IL-6 after exercise is well documented in the literature [39–41]. However, there was no significant difference in plasma IL-6 concentration between N-3 and PLA group. This finding is in accordance with the findings by Tarbinian et al. [41], who found no significant difference between groups in plasma IL-6 concentration immediately post-exercise.

In a manner similar to plasma IL-6, there were no differences in post-EIMD plasma TNF-α concentration between N-3 and PLA groups. There is conflicting evidence about the behaviour of TNF-α response after muscle-damaging exercise. Toft et al. [42] have shown that plasma TNF-α was elevated after prolonged exercise, others did not observe any change [43] and others recorded a decrease in the TNF-α [44]. In the study by Lenn et al. [45], TNF-α was not significantly increased, which is a similar result with the present study, where no significant increase was demonstrated in plasma TNF-α concentration. This could be due to a feedback mechanism, that IL-6 inhibits TNF-α [43]. Thus, it may be that plasma TNF-α is not an optimal marker to quantify EIMD-induced inflammation.

### Functional measurements

We report a significant change in VAS pain score following EIMD in both groups, further evidence that the exercise protocol used caused significant muscle damage. More specifically, participants’ pain perception peaked immediately post-EIMD and remained elevated at 24–48 h post-EIMD, which is consistent with other findings [1, 46]. Our study demonstrated a significant difference in perceived muscle soreness between groups at 24 h post-EIMD, suggesting N-3 may have experienced less pain compared to PLA group at this point. Previous studies [17, 18, 47] also found significant differences in DOMS between groups following EIMD, with the fish oil group having reduced muscle soreness. On the contrary, Jakeman et al. [17] an acute dose of n-3 PUFA immediately after a muscle-damaging exercise, demonstrated similar muscle soreness between groups. The absence of effect on DOMS might be due to the acute supplementation dose following exercise and is insufficient to change muscle phospholipid content [48, 49] relative to the 4 weeks of supplementation used here.

Subsequent exercise performance is significantly affected by EIMD and its symptoms [2]. The loss of muscle force is considered the most valid indirect measurement of muscle damage [50]. As expected, and when observing a large effect size, the leg strength significantly decreased immediately post-EIMD in both groups compared with pre-EIMD values. However, there were no significant differences in MVIC between groups nor was any interaction effect observed, suggesting that levels of muscle damage were unchanged by n-3 consumption. These findings match both those of Gravina et al. [51], who reported no impact of 4 weeks of n-3 supplementation on leg strength, despite a higher dose (equivalent of 0.1 g/kg/day of supplement in capsules, 1000 mg n-3 PUFA per capsule, with a mean intake of 7 ± 2 capsules per day) and of Gray et al. [20] who showed no group effect, with a longer duration (6 weeks) of n-3 supplementation. In addition, a very recent study by Ramos-Campo et al. [52], examining muscle damage after eccentric exercise, found no significant difference in strength deficit between the supplementation and placebo group following a 10-week n-3 supplementation. Therefore, the implications of the findings from the previous studies and ours are that n-3 supplementation does not have significant positive effects on muscle strength recovery.

As a secondary measure of muscle function, we examined cycling peak power, with no significant difference between groups. However, the PLA group demonstrated a significant suppression in peak power at 24 h following EIMD relative to pre, while there was no change in N-3 group relative to pre-EIMD. Decreased cycling peak power output 24 h following EIMD is in line with previous research [53]. The potential for preservation of voluntary peak power output will be of interest to athletes where repeated maximal powerful performance is required, which is reinforced by differences in perceived pain at this timepoint.

In addition, we examined participants’ hydration status before EIMD to ensure they began exercise euhydrated, to prevent hydration being a cofounding factor for exercise performance. We also assessed n-3 intake using a 48-h food diary at pre- and post-supplementation period. No difference in n-3 intake was noted between groups prior to supplementation. As it would be expected, there was an increase in n-3 intake in the N-3 group relative to the PLA group after supplementation.

### Limitations, recommendations and future directions

Some potential limitations of the present study should be acknowledged. Low statistical power due to the modest sample size played a role in limiting the significance of the statistical comparisons conducted. The strict inclusion criteria as well as the downhill running task performance made recruitment for participants difficult. Additional blood biomarkers, such as myoglobin and C-reactive protein, may also provide further information in future studies on muscle damage. A clearer picture on change in muscle function could involve examining n-3 supplementation and muscle damage considering multiple functional measurements, such as MVC torque at multiple joint angles, ROM, limb swelling and/or jump height. Measurements of muscle function should be used in combination with indirect plasma markers to provide more reliable evidence in assessing the magnitude of muscle damage. Ideally, directly measuring muscle damage from muscle biopsies would be optimal, albeit highly invasive. Additionally, future studies may consider taking blood samples at additional acute time points, such as 1, 3, 6 or 12 h after the muscle-damaging exercise. By doing so, we might have observed an acute inflammatory response difference between groups, as has been observed elsewhere [17]. Participants’ diets were not explicitly controlled during the 4-week loading period, however, a 48-h food diary was recorded immediately before supplementation period and repeated 48 h before supplementation finished (immediately before EIMD); results of which suggested participants did not change their habitual macronutrient or total caloric intake. Future studies may consider a method to control participants’ food intake, such as providing pre-packaged meals or recording complete food diaries throughout both supplementation period and recovery phase. However, this would incur both significant cost and require participants to have a greater commitment to these methods. In an attempt to isolate the effect of n-3 supplementation, collagen was chosen as the placebo in this study to avoid manipulation of n-6/n-3 ratio. There is no evidence in the literature that collagen has a pro or anti-inflammatory effect, and therefore, it would not oppose the action of n-3 supplementation. Whereas, other reports have utilized corn oil as a placebo control which is high in n-6, and thus may not represent a true placebo [11, 54].

## Conclusion

Overall, these findings support the hypothesis that 4 weeks of 3 g/day n-3 supplementation may attenuate minor aspects of EIMD, as observed in DOMS and peak power. Typically, no significant differences were noted between groups, however, it was observed a blunted inflammatory response immediately after eccentric exercise and a decreased CK activity at 24 h following muscle-damaging exercise in N-3 group. There were also no significant differences in leg strength between groups indicating that n-3 supplementation will have limited impact on muscle function and subsequent performance. Whilst not improving performance, these findings may have relevance to soreness-associated exercise avoidance.

## Supplementary Information

**Additional file 1.**

**Additional file 2.**

**Additional file 3.**

## Data Availability

The datasets generated and analysed during the current study are available as supplementary material from the corresponding author on reasonable request.

## Abbreviations

ANOVA: Analysis of variance
AA: Arachidonic acid
BIA: Bioelectrical impedance analysis
BMI: Body mass index
CK: Creatine kinase
CV: Coefficient of variation
DHA: Docosahexaenoic acid
DOMS: Delayed-onset muscle soreness
EIMD: Exercise-induced muscle damage
ELISA: Enzyme-linked immunosorbent assay
EPA: Eicosapentaenoic acid
Hb: Haemoglobin
Hct: Haematocrit
HR: Heart rate
IL-1ra: Interleukin-1 receptor
IL-6: Interleukin-6
IL-10: Interleukin-10
MVIC: Maximal voluntary isometric contraction
n-3 PUFA: Omega-3 polyunsaturated fatty acids
N-3: Omega-3 interventional group
PAR-Q: Physical activity readiness questionnaire
PLA: Placebo group
Post-EIMD: Following exercise-induced muscle damage
Pre-EIMD: Before exercise-induced muscle damage
ROM: Range of motion
ROS: Reactive oxygen species
RPE: Rating of perceived exertion
RPM: Revolutions per minute
SD: Standard deviation
TNF–α: Tumor necrosis factor–alpha
USG: Urine specific gravity
VAS: Visual analogue scale
V̇O_2_: Absolute oxygen consumption
V̇O_2_max: Maximal oxygen consumption
Vtest: Downhill running speed
WURSS: Wisconsin upper respiratory symptom survey
