# Supplementary figures and images for: The effect of Omega-3 polyunsaturated fatty acid supplementation on exercise-induced muscle damage
Source: J Int Soc Sports Nutr. 2021 Jan 13;18:9. doi: 10.1186/s12970-020-00405-1 (PMC7807509; doi:10.1186/s12970-020-00405-1)

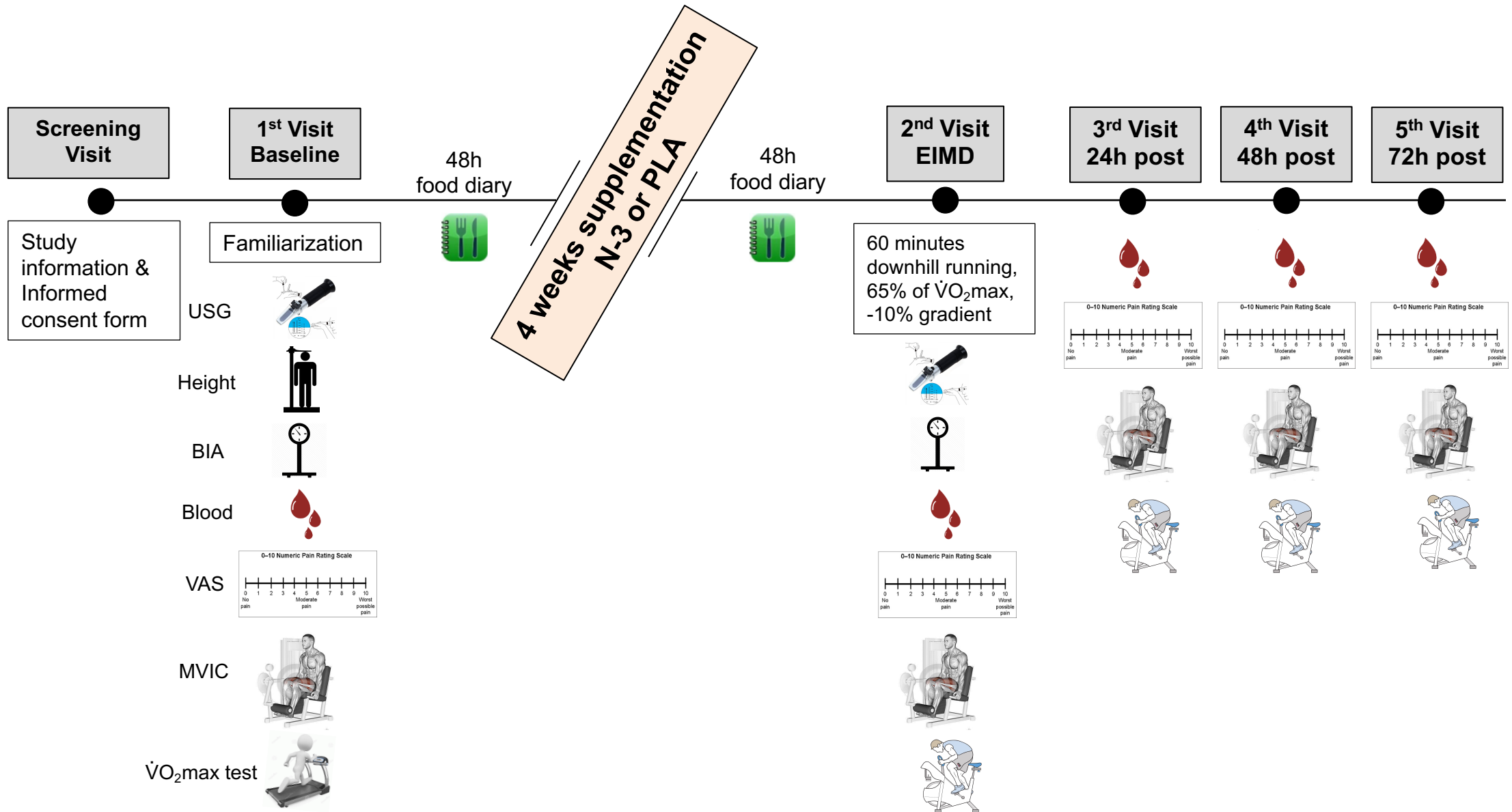

Supplement: Supplementary file 1 — Additional file 1. [file 12970_2020_405_MOESM1_ESM.pdf]

A)

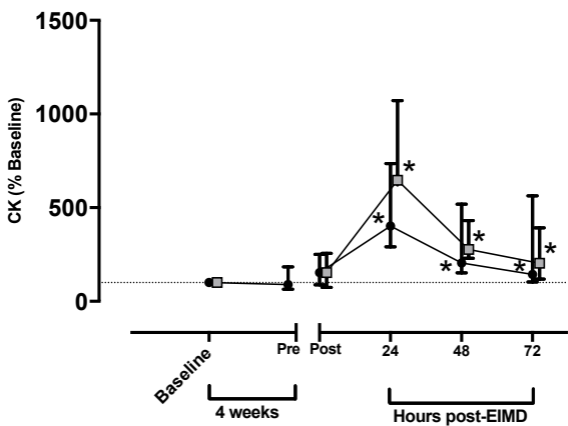

B)

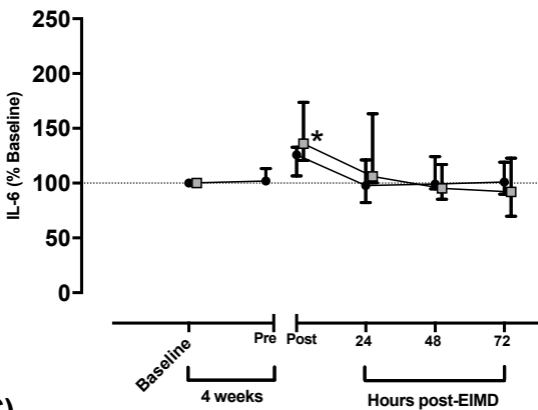

C)

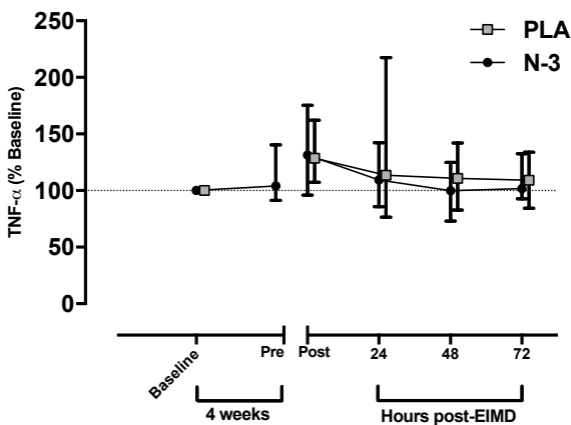

Supplement: Supplementary file 2 — Additional file 2. [file 12970_2020_405_MOESM2_ESM.pdf]

A)

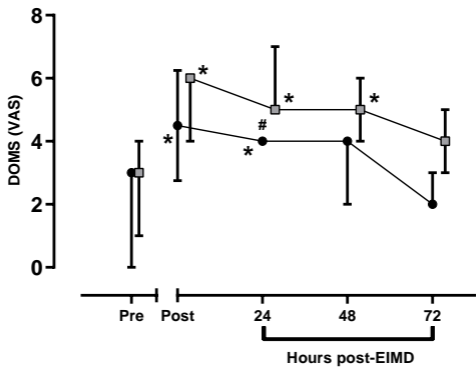

B)

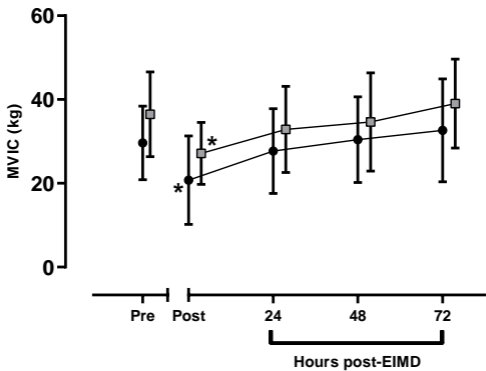

C)

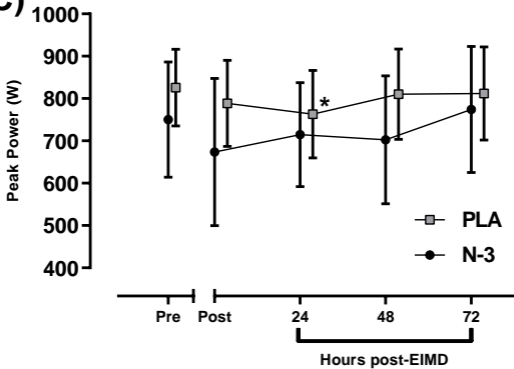

Supplement: Supplementary file 3 — Additional file 3. [file 12970_2020_405_MOESM3_ESM.pdf]
